# Esporte em Tempos de Covid-19: Alerta ao Coração

**DOI:** 10.36660/abc.20200652

**Published:** 2020-09-18

**Authors:** Marcos Perillo, Ricardo Contesini Francisco, Thiago Ghorayeb Garcia, Mateus Freitas Teixeira, Bruno Bassaneze, Lorena Christine Araújo de Albuquerque, Rodrigo Otávio Bougleux Alô, Clea Colombo, Nabil Ghorayeb

**Affiliations:** 1 Instituto Dante Pazzanese de Cardiologia São Paulo SP Brasil Instituto Dante Pazzanese de Cardiologia, São Paulo, SP - Brasil; 2 Universidade Federal Fluminense Niterói RJ Brasil Universidade Federal Fluminense, Niterói, RJ - Brasil; 3 Clube de Regatas Vasco da Gama Rio de Janeiro RJ Brasil Clube de Regatas Vasco da Gama, Rio de Janeiro, RJ - Brasil; 4 Hospital do Coração São Paulo SP Brasil Hospital do Coração, São Paulo, SP - Brasil; 5 Hospital Geral de São Mateus São Paulo SP Brasil Hospital Geral de São Mateus, São Paulo, SP - Brasil; 6 Faculdade de Medicina São Leopoldo Mandic Campinas SP Brasil Faculdade de Medicina São Leopoldo Mandic, Campinas, SP - Brasil

**Keywords:** COVID-19, Betacoronavírus/complicações, Doenças Cardiovasculares/complicações, Esporte, Atleta, Miocardite

## Introdução

Competições esportivas estavam a pleno vapor em 2019, consagrando campeões do ano que findava ou sendo planejadas para suas emocionantes etapas que viriam a seguir. O ano de 2020 traria novos capítulos ao esporte mundial. Porém, o surgimento do novo coronavírus (SARS-CoV-2) mudou o panorama da prática esportiva. Em março de 2020, após declaração da pandemia pela COVID-19, o isolamento social foi adotado por diversos governos, consequentemente proibindo a realização de eventos esportivos. A retomada do esporte vem sendo discutida à medida que as autoridades públicas iniciam o processo de relaxamento do isolamento social, cabendo à sociedade médica propor como melhor avaliar os atletas infectados pela nova doença e determinar a segurança da prática esportiva. A escassez de dados clínicos e epidemiológicos em relação ao acometimento cardíaco nos casos oligossintomáticos, não hospitalizados, da COVID-19, além da incerteza acerca dos desfechos de longo prazo de eventual lesão cardíaca atribuída à doença, fazem da elaboração de recomendações um desafio, sujeitas a mudanças conforme adquirido melhor entendimento da doença.

### Coração e COVID-19

Uma das principais características da COVID-19 é o seu alto poder de contágio e rápida disseminação, além de possível acometimento cardíaco, visto em até 22% dos pacientes hospitalizados,^1^ com taxa de mortalidade até 4,5 vezes maior em cardiopatas.^2^ Após complicações respiratórias e sepse, doenças cardiovasculares são a 3ª causa de morte associada à COVID-19.^3,4^ Em comparação às demais infecções virais associadas à miocardite, estas apresentam taxas bem menores (<1%) de acometimento cardiovascular.^5^ Em estudo com coelhos, foi demonstrado o desenvolvimento de cardiomiopatia dilatada biventricular, hipertrofia, fibrose miocárdica e miocardite em análise histopatológica^6^ e, em seres humanos, o RNA viral foi encontrado no músculo cardíaco em até 35% dos casos de uma série de autópsias,^7^ A COVID-19 pode se manifestar de diversas maneiras, assintomática, sintomática leve (não debilitante), moderada (debilitante) ou grave (hospitalizada)^8^ e desta forma, a apresentação clínica do atleta durante sua avaliação para retorno ao treinamento ou competição pode ser frustrada, não levantando suspeita quanto ao acometimento cardiovascular.

### Lesão Miocárdica

Sinais de lesão miocárdica como o aumento de marcadores de necrose miocárdica, principalmente da troponina, apresenta-se em 8 a 12% dos casos em geral e em até 33% dos pacientes críticos.^8^ Porém, o acometimento miocárdico pode ser assintomático. Os mecanismos de lesão podem ser comuns a qualquer infecção grave, como resposta inflamatória exacerbada, mas também por ação direta viral no tecido cardíaco. Alguns estudos colocam em xeque a capacidade do vírus em gerar lesão direta aos miócitos por não detectá-lo nestas células, levando a crer que sua agressão decorra da combinação de vários fatores, como a resposta inflamatória exacerbada e acometimento microvascular,^9-11^ o que pode gerar coagulação intravascular disseminada, trombose e infarto de grandes e pequenos vasos.^12^ O SARS-CoV-2 infecta células humanas através de sua ligação à enzima conversora da angiotensina 2 (ECA2), consequentemente aumentando os níveis de angiotensina II e seus efeitos deletérios em células onde a expressão do receptor da ECA2 é maior, como nos cardiomiócitos, fibroblastos e pericitos, células localizadas na microvasculatura cardíaca, externas ao endotélio capilar e venular, com relevante papel na microcirculação miocárdica.^9^

### Miocardite

Há grande preocupação quanto à ocorrência de miocardite nos atletas expostos, pois sem adequada avaliação cardiológica especializada, podem ser submetidos durante a fase subaguda ou crônica da doença a volume e intensidade de exercício capazes de desencadear arritmias malignas durante ou mesmo após o esforço. Estima-se que 7 a 20% das mortes súbitas em atletas jovens seja devido a miocardite,^13^ e seu diagnóstico por vezes requer exames complementares, além do exame clínico e eletrocardiográfico. Estudos reportam maior taxa de eventos cardíacos em atletas com maior área de realce tardio à ressonância magnética cardíaca (RMC), mesmo apresentando avaliação ecocardiográfica normal.^14,15^ A miocardite é clinicamente heterogênea, sendo um dos seus principais achados o desenvolvimento ou piora de disfunção ventricular após infecção viral. Dados de uma análise de 150 pacientes de Wuhan na China indicam uma provável incidência de 7% de miocardite em pacientes com COVID-19.^16^ Na fase crônica da miocardite, entre 2 e 12 semanas, ocorre infiltração linfocitária, perpetuando lesão aos miócitos pela resposta imune humoral, e o interstício recebe intensa deposição de colágeno, levando à formação de fibrose, podendo evoluir com dilatação, disfunção e insuficiência miocárdica.^17^

### Retorno

Ao não se considerar o potencial dano miocárdico pela COVID-19, o atleta recuperado com sorologia IgG positiva poderá ser liberado, conforme protocolos institucionais, para o retorno de suas atividades. Porém, conforme bem descrito na literatura, recomenda-se que indivíduos diagnosticados com miocardite sejam afastados por no mínimo 3 a 6 meses.^17^ Dito isto, mesmo após a recuperação, sequelas pró-arrítmicas podem ameaçar o atleta e a avaliação sistematizada é crucial para a adequada estratificação de risco, evitando não somente desfechos adversos como também período desnecessário de afastamento e consequente queda de rendimento e habilidade.^17^

A cinética do *clearance* viral da COVID-19 é alvo de diversos estudos e o tempo de permanência do vírus no organismo ainda não é conhecido, podendo ser detectado RNA viral por mais de 2 meses após início dos sintomas, a despeito de anticorpos detectados nas primeiras 2 semanas.^18^ Deste modo, apesar de postularem que esta positivação prolongada se dê por resquícios de RNA viral, ainda é desconhecido se atletas com anticorpos IgG positivo, considerados imunes, poderiam ou não ser fonte de transmissão do vírus ao seus contactantes e até quando a janela de excreção deste vírus duraria. Logo, um período mínimo de 14 dias de isolamento após infecção confirmada se faz necessário, sendo prudente a avaliação para retorno das atividades esportivas somente após 7 dias assintomáticos.

**Figura 1:**
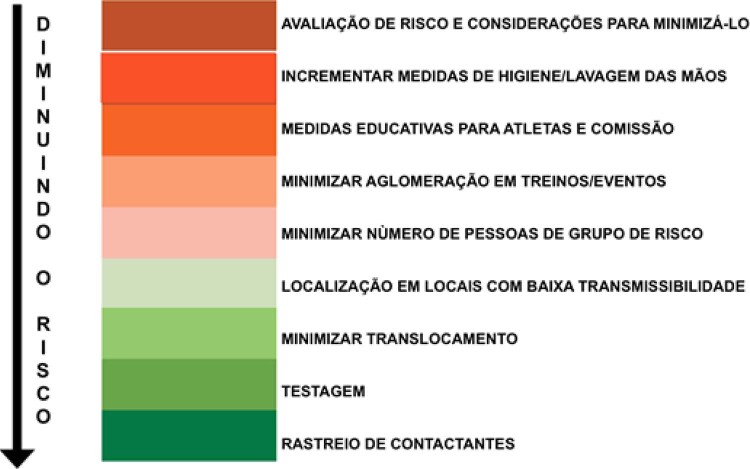
– Medidas para minimizar risco de disseminação da COVID-19 em eventos esportivos. Carmody S, Murray A, Borodina M, et al. When can professional sport recommence safely during the COVID-19 pandemic? Risk Assessment and factors to consider, Posted April 30, 2020.BJSM.

Organizações esportivas de inúmeros países iniciaram a retomada de treinos coletivos e mesmo competições, como o futebol, apesar de evidências limitadas, propondo protocolos de testagem ampla, períodos de afastamento e exames a serem realizados em caso de contato comprovado com o SARS-CoV-2. Entre os variados protocolos, evidencia-se a falta de consenso quanto à obrigatoriedade e maneira ideal de avaliação cardiovascular pós-infecção. A OMS destaca 5 fatores determinantes de risco à realização de eventos esportivos:^19^ (1) se o evento terá sede em país com transmissão local da COVID-19; (2) se haverá sede única ou múltiplas; (3) se atletas e espectadores serão de países com transmissão ativa da doença; (4) se participantes pertencem a grupos de risco em grande número (ex.: maiores de 65 anos ou portadores de comorbidades); (5) se a competição envolve modalidades de alto risco de disseminação da COVID-19 (ex.: esportes de contato). A figura 1 ilustra medidas propostas para minimizar o risco de disseminação em eventos esportivos.

### Avaliação Cardíaca

A avaliação somente por exame clínico, ECG e biomarcadores pode ser insuficiente para diagnóstico de miocardite, pois atletas podem apresentar-se oligossintomáticos, sem novas alterações eletrocardiográficas e com níveis basais elevados de troponina devido ao treinamento, não havendo correlação com nível de realce tardio pela ressonância magnética cardíaca (RMC).^20^ Em avaliação de 670 casos suspeitos clínicos de miocardite, a presença de realce tardio nem sempre era acompanhada de alterações eletrocardiográficas.^21^ O uso de métodos de imagem é essencial nesta avaliação, pois acrescenta dados como disfunção ventricular, alteração de contratilidade segmentar e derrame pericárdico pelo ecocardiograma^17^ e, além destes, realce tardio e edema pela RMC^21^(Tabela 1).

**Tabela 1:** – Achados em exames complementares sugestivos de miocardite

ACHADOS SUGESTIVOS DE MIOCARDITE	
Eletrocardiograma	Alterações novas comparativas ao ECG prévio à pandemia, como: baixa voltagem, bloqueio atrioventricular, arritmias, infradesnivelamento do segmento PR, bloqueio de ramo, alteração do segmento ST, inversão de onda T além de V1-V2 em caucasianos e V1-V4 em afrodescendentes
Teste de esforço	Alteração do segmento ST, arritmias, resposta cronotrópica/hemodinâmica inadequada, intolerância ao esforço, queda de capacidade funcional, sintomas cardiovasculares
Ecocardiograma	Disfunção ventricular, alteração de contratilidade segmentar, derrame pericárdico, dilatação de câmaras
Holter 24 horas	Arritmias, bloqueios atrioventriculares, alteração do segmento ST
Ressonância magnética cardíaca	Disfunção ventricular, alteração de contratilidade segmentar, edema miocárdico, realce tardio

Para melhor acurácia diagnóstica, estratificação de risco e seguimento, a RMC deve ser utilizada na suspeita de miocardite pois, além de melhor avaliar a função ventricular, também é capaz de caracterizar o tecido cardíaco, detectando a presença de edema e fibrose^17^. Por este método, deve-se atentar para além da presença de realce tardio, associada à probabilidade duas vezes maior de eventos cardíacos maiores, sua localização, distribuição e padrão. Associação maior com eventos cardíacos adversos foi demonstrada com o padrão de fibrose de parede septal e miocárdica.^21^

A técnica de mapeamento em T1 e T2 apresenta-se promissora ao conseguir avaliar edema e expansão extracelular em diferentes estágios da doença, melhorando a acurácia da avaliação, principalmente após 2 semanas, quando T2 pode normalizar isoladamente. ^17^ A expansão de volume celular, avaliada pelo mapeamento em T1, quando maior que 10%, se associou a risco quatro vezes maior de morte, mas ainda é um desafio diferenciar o achado entre inflamação ativa e fibrose crônica por este método.^21^

### Proposta de Avaliação Cardiológica em Atletas Acometidos pela Covid-19

**Figura 2:**
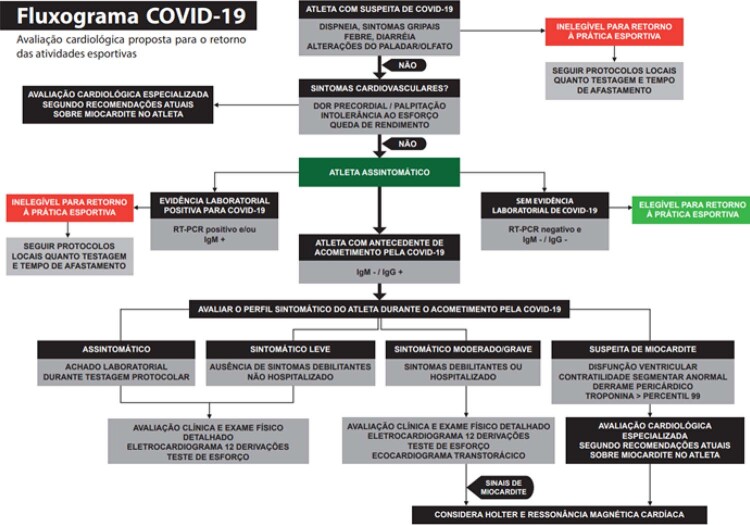
– Fluxograma de avaliação cardiológica do atleta+

A implementação ou não deste protocolo é de responsabilidade das organizações, federações, clubes e entidades médicas que, ao avaliar a exequibilidade, levarão em consideração a realidade local e institucional, em termos de acessibilidade e custos. O fluxograma proposto (Figura 2) sistematiza a avaliação desde a testagem sorológica até a conduta em casos suspeitos de miocardite por COVID-19, que deverá seguir as recomendações atuais sobre miocardite no atleta.^22^ Classifica-se como caso sintomático leve aquele tratado em domicílio e que não desenvolveu sintomas debilitantes, dispneia, dor torácica ou pneumonia. Neste caso, devido à ausência de sintomatologia importante, considera-se que não houve resposta inflamatória significativa e seus potenciais efeitos deletérios, sendo menos provável a ocorrência de miocardite. Indivíduos que apresentaram pneumonia ou outros sintomas debilitantes, contudo também tratados em domicílio, são considerados sintomáticos moderados e, havendo internação hospitalar, considerados sintomáticos grave. Os perfis moderado e grave demandam avaliação em busca de sinais de miocardite, assim como nos casos com histórico ou persistência de sintomas cardiovasculares.

Considera-se elegível para retomada imediata das atividades somente o atleta sem evidência de contato com o SARS-CoV-2 (RT-PCR, IgM e IgG negativos). Em atletas atualmente assintomáticos, mas com comprovação laboratorial da COVID-19 ou histórico de sintomas sugestivos, o perfil sintomático durante a infecção deverá ser avaliado e as medidas a seguir são recomendadas (Tabela 2):

**Tabela 2:** – Perfil sintomático do atleta previamente acometido pela COVID-19

PERFIL SINTOMÁTICO DO ATLETA ACOMETIDO PELO COVID-19			
Assintomático	Sintomático leve	Sintomático moderado/grave	Suspeita de miocardite
Achado laboratorial durante testagem protocolar	Atleta não hospitalizado, sem sintomas debilitantes	Atleta hospitalizado ou com sintomas debilitantes	Sintomas cardiovasculares ou alterações sugestivas em exames complementares
Avaliação clínica e exame físico detalhados em busca de sinais e sintomas cardiovasculares avaliação eletrocardiográfica comparativa a exame anterior à infecção teste de esforço em busca de alterações sugestivas de acometimento cardíaco		Completa avaliação clínico-laboratorial por especialista, incluindo ECG, teste de esforço, ecocardiograma transtorácico, e considerar imagem por ressonância magnética cardíaca e Holter 24 na suspeita de miocardite	Avaliação cardiológica especializada, conforme recomendações atuais sobre miocardite no atleta

1. Assintomático/sintomático leve: avaliação clínica e exame físico detalhados em busca de sinais e sintomas cardiovasculares, avaliação eletrocardiográfica comparativa a exame anterior à infecção e teste ergométrico em busca de alterações sugestivas de acometimento cardíaco;
2. Sintomático moderado/grave: completa avaliação clínico-laboratorial por especialista, incluindo ECG, teste ergométrico, ecocardiograma transtorácico, e considerar imagem por ressonância magnética cardíaca e Holter 24h na suspeita de miocardite;
3. Suspeita de miocardite: associada à completa avaliação clínico-laboratorial por especialista, a imagem por ressonância magnética cardíaca deve ser solicitada para diagnóstico e estratificação de risco.

Em caso de achados clínicos sugestivos de miocardite ou **≥**1 exame complementar alterado em qualquer perfil, o atleta deve ser avaliado por especialista e submetido a seguimento conforme diretrizes atuais de miocardite.

## Conclusão

A retomada do esporte não precedida por avaliação do sistema cardiovascular dos atletas acometidos pela COVID-19 parece imprudente, expondo-os ao risco de morte súbita. Ainda não há dados conclusivos quanto à segurança da prática esportiva de alto rendimento nesta população específica pois, ao serem expostos a esforço intenso, podem apresentar efeitos adversos de eventual lesão miocárdica. Eventos cardíacos podem ocorrer durante partidas, treinos ou mesmo em repouso, o que acarreta grande responsabilidade aos clubes e organizações esportivas, que devem resguardar a si e aos seus integrantes, promovendo adequado rastreio de sequelas cardíacas, principalmente a miocardite. Destaca-se a necessidade de estudos que demonstrem análises de dados provenientes de exames clínicos e complementares desta população, gerando assim possibilidade de protocolos realmente seguros. Até o surgimento destes, deve-se considerar as evidências já existentes quanto à miocardite no atleta.

## References

[1] 1. Phelan D, Kim JH, Chung EH. A Game Plan for the Resumption of Sport and Exercise After Coronavirus Disease 2019 (COVID-19) Infection. JAMA Cardiol,2020; {Cited in 2020 may] Available from: https://jamanetwork.com/journals/jamacardiology/fullarticle/2766124 10.1001/jamacardio.2020.2136 32402054

[2] 2. Wu Z, McGoogan JM. Characteristics of and Important Lessons From the Coronavirus Disease 2019 (COVID-19) Outbreak in China: Summary of a Report of 72 314 Cases From the Chinese Center for Disease Control and Prevention. JAMA. 2020 Feb 24 online.ahead of print 10.1001/jama.2020.2648 32091533

[3] 3. Zhou F, Yu T, Du R, Fan G, Liu Y, Liu Z, et al. Clinical course and risk factors for mortality of adult inpatients with COVID-19 in Wuhan, China: a retrospective cohort study. Lancet. 28 de 2020;395(10229):1054–62. 10.1016/S0140-6736(20)30566-3 PMC7270627 32171076

[4] 4. Chen T, Wu D, Chen H, Yan W, Yang D, Chen G, et al. Clinical characteristics of 113 deceased patients with coronavirus disease 2019: retrospective study. BMJ. 26 de 2020;368:m1091. 10.1136/bmj.m1091 PMC7190011 32217556

[5] 5. Fung G, Luo H, Qiu Y, Yang D, McManus B. Myocarditis. Circ Res. 5 de fevereiro de 2016;118(3):496–514. 10.1161/CIRCRESAHA.115.306573 26846643

[6] 6. Alexander LK, Small JD, Edwards S, Baric RS. An experimental model for dilated cardiomyopathy after rabbit coronavirus infection. J Infect Dis. novembro de 1992;166(5):978–85. 10.1093/infdis/166.5.978 PMC7109931 1328411

[7] 7. Oudit GY, Kassiri Z, Jiang C, Liu PP, Poutanen SM, Penninger JM, et al. SARS-coronavirus modulation of myocardial ACE2 expression and inflammation in patients with SARS. Eur J Clin Invest. julho de 2009;39(7):618–25. 10.1111/j.1365-2362.2009.02153.x PMC7163766 19453650

[8] 8. Tomasoni D, Italia L, Adamo M, Inciardi RM, Lombardi CM, Solomon SD, et al. COVID 19 and heart failure: from infection to inflammation and angiotensin II stimulation. Searching for evidence from a new disease. Eur J Heart Fail. 15 de maio de 2020; 10.1002/ejhf.1871 PMC7273093 32412156

[9] 9. Chen L, Li X, Chen M, Feng Y, Xiong C. The ACE2 expression in human heart indicates new potential mechanism of heart injury among patients infected with SARS-CoV-2. Cardiovasc Res. 01 de 2020;116(6):1097–100. 10.1093/cvr/cvaa078 PMC7184507 32227090

[10] 10. Guzik TJ, Mohiddin SA, Dimarco A, Patel V, Savvatis K, Marelli-Berg FM, et al. COVID-19 and the cardiovascular system: implications for risk assessment, diagnosis, and treatment options. Cardiovasc Res.2020;16(10):1666-87. 10.1093/cvr/cvaa106 PMC7197627 32352535

[11] 11. Varga Z, Flammer AJ, Steiger P, Haberecker M, Andermatt R, Zinkernagel AS, et al. Endothelial cell infection and endotheliitis in COVID-19. Lancet. 02 de 2020;395(10234):1417–8. 10.1016/S0140-6736(20)30937-5 PMC7172722 32325026

[12] 12. Hendren NS, Drazner MH, Bozkurt B, Cooper LT. Description and Proposed Management of the Acute COVID-19 Cardiovascular Syndrome. Circulation. 16 de abril de 2020; 10.1161/CIRCULATIONAHA.120.047349 PMC7314493 32297796

[13] 13. Maron BJ, Haas TS, Ahluwalia A, Murphy CJ, Garberich RF. Demographics and Epidemiology of Sudden Deaths in Young Competitive Athletes: From the United States National Registry. Am J Med. novembro de 2016;129(11):1170–7. 10.1016/j.amjmed.2016.02.031 27039955

[14] 14. Schnell F, Claessen G, La Gerche A, Bogaert J, Lentz P-A, Claus P, et al. Subepicardial delayed gadolinium enhancement in asymptomatic athletes: let sleeping dogs lie? Br J Sports Med. janeiro de 2016;50(2):111–7. 10.1136/bjsports-2014-094546 26224114

[15] 15. Zorzi A, Perazzolo Marra M, Rigato I, De Lazzari M, Susana A, Niero A, et al. Nonischemic Left Ventricular Scar as a Substrate of Life-Threatening Ventricular Arrhythmias and Sudden Cardiac Death in Competitive Athletes. Circ Arrhythm Electrophysiol. 2016;9(7):e004229 10.1161/CIRCEP.116.004229 PMC4956679 27390211

[16] 16. Ruan Q, Yang K, Wang W, Jiang L, Song J. Clinical predictors of mortality due to COVID-19 based on an analysis of data of 150 patients from Wuhan, China. Intensive Care Med. 2020;46(5):846–8. 10.1007/s00134-020-05991-x PMC7080116 32125452

[17] 17. Eichhorn C, Bière L, Schnell F, Schmied C, Wilhelm M, Kwong RY, et al. Myocarditis in Athletes Is a Challenge: Diagnosis, Risk Stratification, and Uncertainties. JACC Cardiovasc Imaging. fevereiro de 2020;13(2 Pt 1):494–507. 10.1016/j.jcmg.2019.01.039 31202742

[18] 18. Liu W-D, Chang S-Y, Wang J-T, Tsai M-J, Hung C-C, Hsu C-L, et al. Prolonged virus shedding even after seroconversion in a patient with COVID-19. J Infect. 2020;81(2):318-56. 10.1016/j.jinf.2020.03.063 PMC7151379 32283147

[19] 19. World Health Organization. (WHO) Considerations for sports federations/sports event organizers when planning mass gatherings in the context of COVID-19: interim guidance. [Cited in 2020 Apr 14]Available from: https://apps.who.int/iris/bitstream/handle/10665/331764/WHO-2019-nCoV-Mass_Gatherings_Sports-2020.1-eng.pdf

[20] 20. Berg J, Kottwitz J, Baltensperger N, Kissel CK, Lovrinovic M, Mehra T, et al. Cardiac Magnetic Resonance Imaging in Myocarditis Reveals Persistent Disease Activity Despite Normalization of Cardiac Enzymes and Inflammatory Parameters at 3-Month Follow-Up. Circ Heart Fail. novembro de 2017;10(11). 10.1161/CIRCHEARTFAILURE.117.004262 29158437

[21] 21. Gräni C, Eichhorn C, Bière L, Murthy VL, Agarwal V, Kaneko K, et al. Prognostic Value of Cardiac Magnetic Resonance Tissue Characterization in Risk Stratifying Patients With Suspected Myocarditis. J Am Coll Cardiol. 17 de outubro de 2017;70(16):1964–76. 10.1016/j.jacc.2017.08.050 PMC6506846 29025553

[22] 22. Ghorayeb N, Stein R, Daher DJ, Silveira AD, Ritt LEF, Santos DFP et al. Atualização da Diretriz em Cardiologia do Esporte e do Exercício da Sociedade Brasileira de Cardiologia e da Sociedade Brasileira de Medicina do Esporte - 2019. Arq Bras Cardiol. 2019; 112(3):326-68.

