# Supplementary material for: Esporte em Tempos de Covid-19: Alerta ao Coração
Source: Arq Bras Cardiol. 2020 Sep 18;115(3):303–7. [Article in Portuguese] doi: 10.36660/abc.20200652 (PMC9363079; doi:10.36660/abc.20200652)
Supplement: Translation

# Translation: Sports in Covid-19 Times: Heart Alert

**Authors:** Marcos Perillo, Ricardo Contesini Francisco, Thiago Ghorayeb Garcia, Mateus Freitas Teixeira, Bruno Bassaneze, Lorena Christine Araújo de Albuquerque, Rodrigo Otávio Bougleux Alô, Clea Colombo, Nabil Ghorayeb

**Affiliations:** 1 Brasil Instituto Dante Pazzanese de Cardiologia, São Paulo, SP - Brasil; 2 Brasil Universidade Federal Fluminense, Niterói, RJ - Brasil; 3 Brasil Clube de Regatas Vasco da Gama, Rio de Janeiro, RJ - Brasil; 4 Brasil Hospital do Coração, São Paulo, SP - Brasil; 5 Brasil Hospital Geral de São Mateus, São Paulo, SP - Brasil; 6 Brasil Faculdade de Medicina São Leopoldo Mandic, Campinas, SP - Brasil

**Keywords:** COVID-19, Betacoronavirus/complications, Cardiovascular Diseases/complications, Sport, Athlete, Myocarditis

## Introduction

Sports competitions were taking place everywhere in 2019, but the novel coronavirus (SARS-CoV-2) has changed the sports scenario around the globe lately. In March 2020, the World Health Organization (WHO) declared COVID-19 (a disease caused by the SARS-CoV-2 virus) pandemic and social isolation was adopted by several governments, prohibiting sporting events. Whereas public authorities have started the process of relaxing social isolation, medical experts have been discussing the best way to evaluate athletes who had COVID-19 aiming a safe return to sports practice. The lack of clinical and epidemiological data regarding cardiac involvement in COVID-19 cases, especially for the oligosymptomatic, non-hospitalized ones, and the unknown consequences of potential cardiac injury, turn this task into a challenge. According to the amount of time passed by, a better understanding of the outcomes and these recommendations may change.

### Heart and COVID-19

One of the main characteristics of COVID-19 is its high contagion power and rapid spread, in addition to the potential cardiovascular involvement, seen in up to 22% of hospitalized patients,^1^ with a mortality rate of up to 4.5 times greater in cardiac patients.^2^ After respiratory complications and Sepsis, cardiovascular disease is the 3^rd^ leading cause of death associated with COVID-19.^3,4^ Comparing with other viral infections that cause myocarditis, these have much lower rates (<1%) of cardiovascular involvement.^5^ A study with rabbits found the development of biventricular dilated cardiomyopathy, hypertrophy, myocardial fibrosis and myocarditis through histopathology.^6^ In humans, the viral RNA was found in the cardiac muscle in up to 35% of the cases of a series of autopsies.^7^ COVID-19 might manifest itself in several ways, asymptomatic, mildly symptomatic (not debilitating), moderately symptomatic (debilitating) or severely symptomatic (hospitalized).^8^ Thus, the clinical presentation of athletes diagnosed with COVID-19 in their assessment to return to play may not raise suspicion of cardiovascular involvement.

### Myocardial Injury

Signs of myocardial injury, such as increased cardiac biomarkers, mainly troponin, are present in 8 to 12% of cases in general and in up to 33% of critically ill patients,^8^ being identified as independent prognostic factor of mortality.^8^ The mechanisms of injury can be common to any severe infection, such as increased inflammatory response, but also by direct action of the virus in the cardiac tissue. Some studies call into question the ability of the virus to generate direct damage to myocytes by not detecting the virus in these cells, leading to the belief that its aggression results from the combination of several factors, such as exacerbated inflammatory response and microvascular involvement,^9-11^ generating disseminated intravascular coagulation, thrombosis and infarction of large and small vessels.^12^ The SARS-CoV-2 infects human cells binding to the angiotensin-converting enzyme 2 (ECA2), consequently increasing angiotensin-II levels and its deleterious effects, where ECA2 receptor expression is greater (cardiomyocytes, fibroblasts and pericytes, cells located in the external part of the capillary and venular endothelium), playing an important role in myocardial microcirculation damage.^9^

### Myocarditis

There is a great concern about the occurrence of myocarditis in athletes and, without proper specialized cardiological evaluation, they might be exposed to high volume and intensity of exercise during the subacute or chronic phase of the disease and, at this stage, malignant arrhythmias may occur during or even after exertion. It is estimated that 7 to 20% of sudden deaths in young athletes are due to myocarditis,^13^ and its diagnosis often requires complementary exams, besides clinical and electrocardiographic examination. Studies report a high rate of cardiac events in athletes with a greater area of late gadolinium enhancement (LGE) on cardiac magnetic resonance imaging (CMR), even with normal echocardiographic evaluation.^14,15^ Myocarditis is clinically heterogeneous, one of its main findings is the development or worsening of ventricular dysfunction after viral infection. Data from an analysis of 150 patients in Wuhan in China, indicates a probable incidence of 7% of myocarditis in patients with COVID-19.^16^ At the chronic phase of myocarditis between the 2^nd^ and 12^th^ week, lymphocytic infiltration occurs, perpetuating myocyte damage by the humoral immune response and the interstice receives intense collagen deposit, leading to the formation of fibrosis, which may lead to dilation, dysfunction and myocardial insufficiency.^17^

### Sports Return

When potential myocardial damage by COVID-19 is not considered, athletes recovered from COVID-19 with positive IgG serology may be eligible to return to their activities, according to institutional protocols. However, as well described in the literature, it is recommended that individuals diagnosed with myocarditis do not practice intense physical activity for at least 3 to 6 months.^17^ Therefore, even after recovery, life-threatening pro-arrhythmic sequelae may exist in the athlete’s heart. Systematic assessment is crucial for adequate risk stratification to avoid unexpected outcomes and an unnecessary period of absence with consequent loss of performance and skills, as well.^17^

The kinetics of viral clearance of COVID-19 has been studied and its remaining period in the human body is still unknown. Detection of SARS-CoV-2 RNA for more than 2 months after the first symptoms and signs may occur, despite antibodies being detected on the 10^th^ day.^18^ Thus, although it is postulated that this delayed detection is due to remnants of viral RNA, it is still unknown whether athletes with positive IgG antibodies, firstly considered immune, could be transmitting the virus to their contacts and for how long. Based on that, a minimum period of 14 days of isolation after infection is necessary, being prudent to evaluate the athlete for resumption only after 7 days without symptoms.

Sports organizations from numerous countries have started to resume collective training and even competitions, such as football, despite limited evidence, proposing wide testing protocols, periods of absence and exams to be performed in case of proven contact with SARS-CoV-2. Among the various protocols, there is no consensus on the mandatory and ideal way of post-infection cardiovascular evaluation. The WHO highlights five risk factors to sporting events organization:^19^ (1) whether the event will be based in a country with local transmission of COVID-19; (2) whether there will be a single or multiple headquarters; (3) whether athletes and spectators will be from countries with active transmission of the disease; (4) whether a large number of participants belong at-risk groups (e.g.: older than 65 years or with comorbidities); (5) whether the competition involves modalities with high risk of COVID-19 spread (e.g.: contact sports). Figure 1 illustrates the measures proposed to minimize the risk of dissemination at sporting events.

**Figure 1:** – Measures to minimize the risk of dissemination of COVID-19 at sporting events. Carmody S, Murray A, Borodina M, et al. When can professional sport recommence safely during the COVID-19 pandemic? Risk Assessment and factors to consider, posted April 30, 2020. BJSM.

### Cardiac Assessment

Assessment limited to clinical examination, ECG and biomarkers might be insufficient for myocarditis diagnosis, because the athletes could be oligosymptomatic without new ECG findings and presenting high baseline troponin levels due to training, showing no correlation with LGE on CMR.^20^ In the evaluation of 670 patients with suspected myocarditis, the presence of LGE was not necessarily accompanied by electrocardiographic abnormalities.^21^ The use of imaging methods is essential in this assessment, giving information about ventricular dysfunction, segmental contractility abnormalities and pericardial effusion by echocardiogram evaluation,^17^ and in addition to these, LGE and edema by CMR^21^(Table 1).

**Table 1:** – Suggestive findings of myocarditis

SUGGESTIVE FINDINGS OF MYOCARDITIS	
Electrocardiogram	New ECG findings: low voltage, AV block, arrhythmia, PR deviation, bundle branch block, ST deviation, T wave inversion beyond V1-V2 in Caucasians and V1-V4 in Afro-Caribbeans
Stress Test	ST deviation, arrhythmia, inadequate chronotropic/hemodynamics response, exercise intolerance, loss of functional capacity, symptoms
Echocardiogram	Ventricular disfunction, wall motion abnormalities, pericardial effusion, dilatation
24-hour Holter	Arrhythmia, AV block, ST deviation
Cardiac Magnetic Resonance Imaging	Ventricular disfunction, wall motion abnormalities, myocardial edema, late gadolinium enhancement

For accurate diagnosis, risk stratification and better follow-up, CMR should be used in the suspicion of myocarditis, because in addition to a better assessment of ventricular function, it is also able to identify abnormalities of cardiac tissue, detecting the presence of edema and fibrosis.^17^ By this method, special attention should be paid to the presence of LGE, associated with a two-fold higher probability of major cardiac events (MACE), as well as their location, distribution and pattern. A greater association with MACE was demonstrated with the pattern of septal and midwall fibrosis.^21^

CMR with T1 and T2 mapping technique is promising while assessing edema and extracellular expansion at different stages of the disease, improving accuracy of the method, especially after 2 weeks when T2 could normalize in isolation.^17^ The expansion of cellular volume, assessed by T1 mapping, when greater than 10%, was associated with a four times greater risk of death, but it is still challenging to differentiate active inflammation from chronic fibrosis by this technique.^21^

### Proposal of Cardiac Evaluation of Athletes Affected by Covid-19

Implementing or not this protocol is the responsibility of organizations, federations, clubs and medical entities, while assessing the feasibility will take into account the local and institutional reality, in terms of accessibility and costs. The proposed flowchart (figure 2) systematizes the evaluation from serological testing to the management of suspected cases of myocarditis by COVID-19, which must be evaluated according to updated recommendations about myocarditis in athletes.^22^ This article defines as mildly symptomatic those treated out of hospital without debilitating symptoms, dyspnea, chest pain or pneumonia. In this case, due to the absence of severe symptoms, it is considered that there was no significant inflammatory response and its potential deleterious effects, which makes the occurrence of myocarditis unlikely. Individuals who had pneumonia or other debilitating symptoms, but were also treated out of hospital, are considered moderately symptomatic, and those treated in hospital are considered severely symptomatic. Both profiles require evaluation for signs of myocarditis, as well in cases with a prior history or persistence of cardiovascular symptoms.

**Figure 2:** – Athletes cardiac evaluation flowchart.

Only athletes without evidence of contact with SARS-CoV-2 (negative RT-PCR, IgM and IgG) are considered eligible for immediate resumption of sports. For those currently asymptomatic with evidence or history of symptoms suggestive of COVID-19, their symptoms profile during the infection must be evaluated and the following measures are recommended (table 2):

**Table 2:** – Symptoms profile

SYMPTOMS PROFILE OF ATHLETES WITH PREVIOUS COVID-19			
Asymptomatic	Mild Symptoms	Moderate/Severe Symptoms	Myocarditis Suspicion
Laboratory finding during protocol testing	Non-hospitalized, without debilitating symptoms	Hospitalized or without debilitating symptoms	Cardiovascular symptoms or suggestive findings in complementary exams
Detailed clinical evaluation and physical examination in search of cardiovascular symptoms and signs Electrocardiographic evaluation comparing to previous exam Stress test in search of suggestive findings of cardiac involvement		Comprehensive clinical and laboratory evaluation by a specialist, including ECG, stress test, transthoracic echocardiogram; cardiac magnetic resonance imaging and 24-hour Holter should be considered in the suspicion of myocarditis	Cardiac evaluation according to updated recommendations about myocarditis in athletes

1. Asymptomatic/mildly symptomatic: detailed clinical evaluation and physical examination in search of cardiovascular signs and symptoms, comparative ECG prior to infection and stress test in search of signs of cardiac involvement;
2. Moderately/severely symptomatic: comprehensive clinical and laboratory evaluation by a specialist, including ECG, stress test, transthoracic echocardiogram; cardiac magnetic resonance imaging and 24-hour Holter should be considered in the suspicion of myocarditis;
3. Suspected myocarditis: associated with comprehensive clinical and laboratory evaluation by a specialist, cardiac magnetic resonance imaging must be performed for diagnosis and risk stratification.

In the case of clinical findings suggestive of myocarditis or ≥1 abnormal complementary exam in any profile, the athlete must be evaluated by a specialist and submitted to follow-up according to existing recommendations about myocarditis in athletes.

## Conclusion

Resumption of sports practice not preceded by adequate pre-participation cardiac evaluation in athletes affected by COVID-19 may be a thoughtless decision which exposes them to the risk of sudden cardiac death. There is still no conclusive data regarding the safety of high-performance sports in this specific population because during vigorous exercise, they might present malignant arrhythmias secondary to myocardial injury with bad outcomes. Sports teams and organizations are responsible for taking care of their athletes and staff, promoting adequate screening for cardiac sequelae (especially myocarditis), because cardiac events can occur during matches, training or even at rest. Further studies are needed to demonstrate data analysis from clinical and complementary exams in this population, which should base the recommendations for a safe return to sports practice. So far, we should consider the risk of myocarditis in athletes with previous diagnosis of COVID-19 and follow the current recommendations for evaluating athletes with myocarditis.
